# Associations between taxi drivers’ aggressive driving behavior and sleep, cognition, and psychological factors in Korea: negative binomial regression analysis

**DOI:** 10.4178/epih.e2024085

**Published:** 2024-10-17

**Authors:** Jong Sun Ok, Hyeongsu Kim, Soo Young An, Mi Young Kim

**Affiliations:** 1 Department of Nursing, Konkuk University College of Biomedical and Health Science, Chungju, Korea; 2 Department of Preventative Medicine, Konkuk University School of Medicine, Chungju, Korea; 3 Department of Emergency Medicine, Konkuk University Medical Center, Chungju, Korea; 4 Department of Nursing, Hanyang University College of Nursing, Seoul, Korea

**Keywords:** Aggressive driving, Sleepiness, Cognitive psychology

## Abstract

**OBJECTIVES:**

Aggressive driving behavior is a significant predictor of traffic accidents. In particular, the driving behavior of taxi drivers is a critical issue that can impact the safety of both drivers and passengers. This study explored the sleep, cognitive, and psychological factors associated with taxi drivers’ aggressive driving behavior.

**METHODS:**

In this descriptive study, a self-report questionnaire was distributed to taxi drivers in Seoul and Gyeonggi Province from August 22, 2022 to December 30, 2022. In all, 992 respondents were analyzed using negative binomial regression.

**RESULTS:**

The mean score for aggressive driving behavior among taxi drivers was 13.76±11.47, with sub-scores of 3.46±3.48 for lapse, 3.31±3.16 for error, and 6.99±5.76 for violation. Contributing factors included sleep disorders, cognitive decline, and psychological factors. A higher score for aggressive driving behavior was associated with an increased severity of insomnia and daytime sleepiness, higher rates of cognitive failure, and elevated levels of depression and stress.

**CONCLUSIONS:**

Our findings highlight the importance of addressing the sleep, cognitive, and psychological factors associated with aggressive driving behaviors among taxi drivers. Further study is needed to evaluate the causal relationship. In addition, it is imperative to develop educational programs and interventions to manage these issues effectively.

## GRAPHICAL ABSTRACT

[Fig f2-epih-46-e2024085]

## Key Message

It is important to predict taxi drivers’ aggressive driving behavior to decrease the traffic accidents that result from such behavior. Previous studies have shown that general and occupational characteristics have a significant impact on aggressive driving behavior, whereas research on the psychological factors is limited. Our comprehensive investigation of the psychological factors as well as the sleep factors and cognitive functions of taxi drivers (as part of a rapidly aging population) showed a significant relationship between these factors and aggressive driving behavior.

## INTRODUCTION

Taxis serve as both public and private transportation modes, positioning taxi drivers as important figures in ensuring the well-being and safety of the public [1]. Environmental factors, such as extended driving periods and traffic congestion, render taxi drivers more susceptible to accidents than the general driving population [2]. In Korea, as of March 2023, there were 248,913 licensed taxi vehicles (164,670 private and 84,243 corporate) with 235,736 taxi drivers (164,670 private and 71,066 corporate) [3].

The population of older adults is rapidly growing in Korea and is expected to enter a super-aging society by 2025. The aging of commercial vehicle drivers is also progressing rapidly. In 2021, of the 740,000 commercial vehicle drivers (bus, taxi, truck), 155,000 (20.8%) were aged ≥ 65 years. In particular, the proportion of elderly drivers in the taxi industry was 39.6% (n=95,000) out of 240,000 taxi drivers [4]. One of the factors accelerating the aging of taxi drivers is the reluctance of young people to work as taxi drivers due to the low wages, long driving hours, and poor working conditions that do not allow for sufficient rest [5]. Taxi drivers also experience night shift work, lack of physical activity, high levels of verbal and physical violence, and increased competition from ride-sharing services [6].

The year 2022 witnessed 12,392 taxi accidents (6,655 corporate and 5,737 private taxis) resulting in 110 fatalities and 17,694 injuries [7]. The primary causes were non-adherence to safe driving protocols in 6,482 cases, traffic signal violations in 1,644 cases, and failure to maintain safe distances in 1,567 cases. As of 2022, the accident rate of taxi drivers aged ≥ 65 years was approximately 5.5% [5].

Previous studies have reported that increasing age affects these various factors. The lack of sleep that accompanies aging has been shown to increase the number of missed responses by drivers in driving simulations [8], and cognitive ability is reported to worsen with age [9]. Psychological factors such as severe driving anxiety [10] and depressive symptoms are closely related to driving cessation among drivers > 70 years of age [11]. In addition, it has been reported that increased driving stress in older drivers is related to deterioration in driving ability [12]. However, previous studies have mainly looked at the personal characteristics and environmental factors related to the driving behavior of taxi drivers [13], while research on sleep problems, cognition, and psychological aspects is limited. People are often reluctant to complain about mental health problems or ask for help due to the stigma related to mental health issues [14].

This study explored the relationship between sleep quality, cognitive function, and psychological factors in taxi drivers and their aggressive driving behaviors within the context of Korea’s transportation culture and work environment. Through this investigation, we hope to establish a foundation for formulating policies to mitigate traffic accidents.

## MATERIALS AND METHODS

### Study design and population

This cross-sectional study investigated the factors related to aggressive driving behavior among taxi drivers, focusing on sleep quality, cognitive function, and psychological health. Participants included 1,023 taxi drivers aged ≥ 30 years in Seoul and Gyeonggi Province who consented to participate in the study and completed the survey voluntarily from August 22, 2022 to December 30, 2022. After excluding 31 individuals with missing responses to key variables, the final study population was 992 participants.

### Variables

#### Dependent variable: aggressive driving behavior of taxi drivers

The aggressive driving behavior of taxi drivers was assessed using the Driving Behavior Questionnaire (DBQ). This questionnaire evaluates lapses, which are absent-minded behaviors primarily affecting the individual; errors, often observational failures that could endanger others; and violations, which are deliberate breaches of safe driving practices [15]. The DBQ, adapted for Korean drivers [16], included 8 questions on errors, 8 on lapses, and 12 on violations. Responses were measured on a 6-point Likert scale, ranging from 0 (“never”) to 5 (“nearly all of the time”). Higher scores indicated a greater risk of aggressive driving behavior. The total score ranged from 0 points to 140 points, with subgroup scores ranging from 0 points to 40 points for lapses and errors, and 0 points to 60 points for violations.

#### Independent variables: sleep, cognition, and psychological factors

Sleep quality was evaluated using the Insomnia Severity Index (ISI) and the Epworth Sleepiness Scale (ESS). The severity of sleep insomnia was measured using the Korean version of the ISI, developed by Bastien et al. [17] and validated by Cho et al. [18], which consists of 7 questions rated on a 5-point Likert scale. Total scores range from 0 to 28, with higher scores indicating more severe insomnia. Insomnia severity was categorized as follows: 0-7 points indicated no clinically significant insomnia, 8-14 points indicated subthreshold insomnia, 15-21 points indicated moderate insomnia, and 22-28 points indicated severe insomnia. The reliability of the tool of Cho et al. [18] was demonstrated by a Cronbach’s alpha of 0.92. The Cronbach’s alpha in this study was 0.88. Daytime sleepiness was assessed using the most recent version of the ESS developed by Johns [19] in 1991. The ESS, a publicly available instrument, includes 8 questions rated on a 4-point Likert scale. Higher scores reflect greater daytime sleepiness. The scoring range is 0-24 points, with the original tool’s reliability shown by Cronbach’s alpha values ranging from 0.73 to 0.88 [19]. The Cronbach’s alpha was 0.88 in this study.

Cognition was assessed using subjective memory decline and cognitive failure measures. Subjective memory decline was evaluated through the Subjective Memory Complaints Questionnaire developed by Youn et al. [20]. This tool includes 14 questions, with 4 addressing overall memory loss and 10 focusing on memory loss in daily activities. A response of “yes” to each question is scored as 1 point, while “no” responses score 0 points. The total score ranged from 0-14 points, with higher scores indicating more severe subjective memory decline. The reliability of the tool at the time of its development was shown by a Cronbach’s alpha of 0.86 [20], and Cronbach’s alpha was 0.82 in this study.

Cognitive failure was measured using the Korean version of the Cognitive Failure Questionnaire developed by Boomsma [21] and later modified and adapted by Lee et al. [22]. This questionnaire includes 25 items that examine the frequency of specific errors, such as forgetting names, overlooking signs, accidentally bumping into people, and being unintentionally distracted. Responses are rated on a 5-point Likert scale from 0 (“never”) to 4 (“always”), reflecting the frequency of cognitive failures experienced over the past 6 months. The score range is 0-100, with higher scores denoting a higher frequency of cognitive failures. In the study by Lee et al. [22], the Cronbach’s alpha was 0.88, and it was 0.96 in the current study.

Psychological factors were assessed using the Korean version of the Depression Anxiety Stress Scale, developed by Henry & Crawford [23] and modified and translated by Jun et al. [24]. This scale includes 21 questions, including 7 for depression, 7 for anxiety, and 7 for stress. Each item is rated on a 4-point Likert scale from 0 (“does not apply at all”) to 3 (“applies very much or most of the time”). The total score ranges from 0 to 63, with higher scores reflecting greater severity of symptoms. At the time of the original tool’s development, the Cronbach’s alpha was 0.91 [23], while in the study by Jun et al. [24] it was between 0.83 and 0.87, and in this study it was 0.96.

#### Adjusted variables: general and driver-related characteristics of taxi drivers

The general characteristics examined included age, sex, body mass index (BMI) (weight in kilograms divided by the height in meters squared), education level, marital status, number of family members living together, personal monthly income, smoking habits, alcohol consumption, hobbies, and the presence and type of comorbidities. The types of comorbidities explored were hypertension (HTN), diabetes mellitus (DM), digestive disorders, disc disease, arthritis, angina and myocardial infarction, cancer, stroke, liver disease, cataracts, heart failure and arrhythmia, thyroid disease, and hyperlipidemia.

The driver-related characteristics assessed included the type of taxi, driving experience, daily driving time and distance, number of night shifts per fortnight, and use and type of car options.

### Statistical analysis

**Figure 1.**
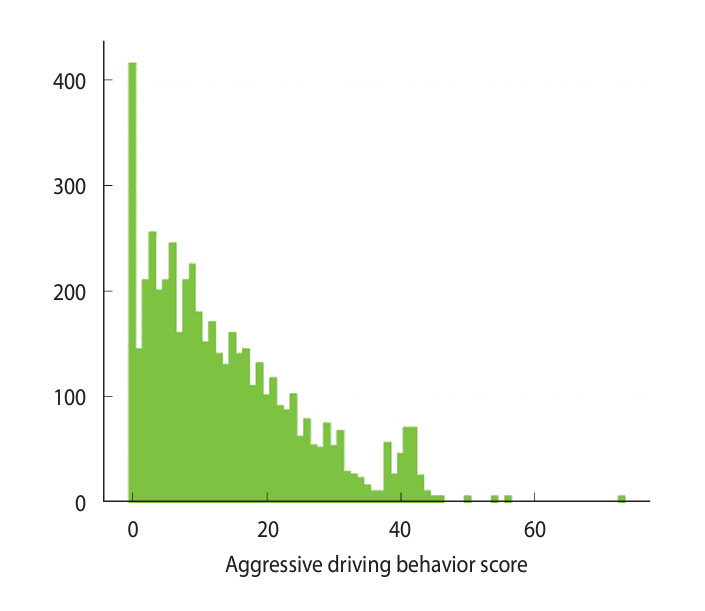
Aggressive driving behavior scores among taxi drivers.

The data were analyzed using R version 4.1.3 (R Foundation for Statistical Computing, Vienna, Austria). The participants’ general characteristics, driving-related characteristics, sleep disorders, cognitive decline, and psychological factors were presented as frequencies, percentages, means, and standard deviations. Negative binomial regression analysis was used to identify factors associated with the aggressive driving behaviors of the taxi driver cohort. This method is suitable for data characterized by skewness towards zero and overdispersion exceeding the mean. In this study, the mean and variance of the aggressive driving behavior scores were 13.76 and 133.68 (Figure 1). Negative binominal regression analysis can reduce the problems of bias and underestimation of standard error and the variance in cases of overdispersion where the variance is greater than the mean [25].

Missing values were handled through multiple imputation, repeating the algorithm 10 times. The model’s goodness-of-fit was evaluated by a log_2_-likelihood of -34,738.48 and an Akaike information criterion of 34,756. All variance inflation factor values used to assess multicollinearity were < 10, indicating no multicollinearity concerns.

### Ethics statement

The study protocol was conducted according to the principles of the Declaration of Helsinki and was approved by the Institutional Review Board of the Clinical Research Ethics Committee of Konkuk University, Seoul, Korea (approval No. 7001355-202404-E-773). Written informed consent was obtained from all participants prior to the survey.

## RESULTS

### General characteristics and driving-related features of the study population

The general and driving-related characteristics of the study population are presented in Table 1. The average age of participants was 59.27±6.84 years, and 98.3% were male. The mean BMI was 24.67±2.77 kg/m^2^. The highest level of education was high school graduation (66.6%), the average household size was 1.60±1.08 members, and 75.7% were married. The predominant monthly income bracket was between 1.50 million Korean won and 1.99 million Korean won. Rates of smoking and drinking were 46.5% and 73.0%, respectively, and 80.0% of participants had hobbies. The prevalence of comorbidities was 56.8%, with HTN, DM, digestive system diseases, disc disorders, and arthritis being the most common (Table 1).

Regarding driving-related characteristics, 70.6% were corporate taxi drivers and 29.4% were private taxi drivers. The average driving experience spanned 197.86±120.56 months, with daily driving times and distances averaging 10.20±2.36 hours and 223.28±67.18 km, respectively. The average number of night shifts over 2 weeks was 5.12±4.87 days. Utilization of driving aids was reported at 47.6%, with the most common being lane-keeping and lane-departure warning systems (87.1%), rear collision-prevention systems (73.9%), and forward collision-prevention systems (46.0%) (Table 1).

### Score of aggressive driving behavior

The overall aggressive driving behavior score among taxi drivers was 13.76±11.47 points. By subgroup, the scores were 3.46±3.48 for lapses, 3.31±3.16 for errors, and 6.99±5.76 for violations. Notably, the error category question, “attempt to overtake someone turning left,” received a particularly high score of 0.95±0.99. In the violations category, the items scoring highest were question 10 (“push in at the last minute,” 0.91±0.71), question 16 (“overtake a slow driver on the inside,” 0.90±0.83), and question 26 (“disregard the speed limit on a motorway,” 0.90±0.79) (Table 2).

### Sleep, cognition, psychological factors

The average ISI score was 4.71±4.11, with 75.5% of participants showing no clinically significant insomnia, 21.9% exhibiting subthreshold insomnia, and 2.6% experiencing moderate insomnia. The daytime sleepiness score averaged 4.74±3.70. For subjective memory decline, the total score was 1.54±2.20, with scores for overall memory loss and daily life memory loss at 0.70±0.86 and 0.84±1.61, respectively. The cognitive failure score was 11.15±10.88. Scores for the psychological factors, depression, anxiety, and stress were 2.05±2.91, 1.84±2.64, and 2.44±3.15, respectively, contributing to a total score of 6.33±8.36 (Table 3).

### Relationships between aggressive driving behavior and sleep, cognition, psychological factors

Negative binomial regression analysis, after adjusting for variables, identified relationships between taxi drivers’ aggressive driving behavior and insomnia severity, daytime sleepiness, cognitive failure, and psychological factors (depression, anxiety, and stress). Specifically, increased insomnia severity and daytime sleepiness, greater cognitive failure, higher levels of depression and stress, and lower levels of anxiety were associated with an increase in aggressive driving behavior (Table 4).

## DISCUSSION

To provide evidence for the formulation of policies that mitigate traffic accidents, this study explored the connection between sleep quality, cognitive function, and psychological factors in taxi drivers and their aggressive driving behavior.

This study showed that higher scores for aggressive driving behavior were associated with more severe insomnia and daytime sleepiness, higher rates of cognitive failure, and elevated levels of depression and stress. We used the 28-item DBQ tool [16], which resulted in a total score for aggressive driving behavior of 13.76 points. In subgroup analysis, the violation score was particularly high at 5.76 points. Although other studies have used the DBQ tool, direct comparison was difficult due to differences in the research subjects and the composition of the questions. Moreover, the aggressive driving behavior scores varied from 5.86 points to 17.39 points [26-28]. Our study found that the aggressive driving behavior scores of the subjects were relatively high. In addition, violation scores varied from 1.47 points to 6.39 points in other studies [26-28], while the violation scores in this study were comparatively high.

Notably, the violation items that had high scores in previous studies, such as “overtake a slow driver on the inside” and “push in at the last minute” [27,29,30] were similar to those in this study. These findings indicate that both taxi drivers and the general driving population frequently engage in speeding and red-light violations, suggesting a commonality in aggressive driving behaviors across different demographics and locations. This finding also corroborates previous reports indicating that taxi drivers often engage in speeding and traffic violations as a strategy to increase their earnings, given that their income is directly tied to the distances they cover [31]. Therefore, a systematic approach is needed to prevent speeding and red-light violations. For example, if the remaining time for a green light is posted so that drivers at an intersection are aware of it, drivers will reduce speeding through lights and prevent red-light violations at intersections. Future transportation policies must consider the factors related to aggressive driving behavior and implement interventions that further reduce it.

Second, an increase in the severity of insomnia and daytime sleepiness was associated with higher scores for aggressive driving behavior, aligning with existing research that links sleep deprivation, poor sleep quality, and subjective drowsiness with impaired driving capability in the general driving population [32]. In particular, sleep deprivation that accompanies aging is reported to increase the number of missed responses by drivers in driving simulation situations [8]. A study in the United States found that more than half of the taxi drivers in New York City reported poorer sleep quality than the general population. Nearly half (48.5%) reported sleeping < 7 hours a night, surpassing the broader population (38.3%) [33]. Lack of sleep causes driver fatigue [34], and driving while drowsy is an all-important cause of traffic accidents [35]. Furthermore, the demanding schedules of taxi drivers compromise their ability to obtain sufficient rest. In particular, taxi drivers who work night shifts experience more severe sleep disturbances than their day shift counterparts [33]. Given the demands of their job, which often requires driving late at night, early in the morning, or for extended periods, taxi drivers are prone to fatigue and drowsiness, increasing their risk of accidents. Accordingly, measures such as ‘restrictions on driving time without rest’ [36] and rest areas where drivers can take a short rest on the highway are being applied as measures to reduce driver fatigue [37]. In addition, policy efforts are needed to ensure that taxi drivers themselves are aware of the seriousness of sleep deprivation and can accurately evaluate their own sleep time and sleep quality.

Third, an increase in cognitive impairment corresponds with higher scores of aggressive driving behavior in this study. Given that cognitive functions tend to deteriorate with age [9], the decline in cognitive abilities among aging taxi drivers appears inevitable. As the probability of mild cognitive impairment increases in adults aged > 50 years [38], it is necessary to include a cognitive function evaluation in health checkups for taxi drivers. Rather than restricting driving based on the degree of cognitive decline, education and counseling programs are needed to strengthen cognitive function.

Last, this study identified that higher scores for depression and stress, coupled with lower scores for anxiety, were associated with increased aggressive driving scores. Previous studies have shown that people with higher levels of anxiety due to social concerns and fear related to accidents or danger are more likely to avoid or pay excessive attention to potential accidents or dangers [39]. Huang et al. [40] reported that as social anxiety increases, aberrant driving behavior decreases. However, it has also been reported that, as driving stress increases in elderly drivers, driving ability deteriorates [12]. In fact, drivers over the age of 70 have serious driving anxiety, so they tend to reduce the number of times and the distance they drive [10]. Depressive symptoms are also closely related to driving cessation in elderly drivers [11]. Recently, there has been increased interest in the driving behavior and mental health of taxi drivers. Driving behavior is a fairly complex behavioral pattern and is influenced by conscious and unconscious actions called cognitive-behavioral characteristics [41]; thus, the need to pay closer attention to the mental health of taxi drivers. The mental health problems of taxi drivers such as depression and anxiety should be recognized as chronic diseases and efforts should be made to manage them at an appropriate level.

This study had several limitations. First, the cross-sectional design limited the ability to establish causality between the identified variables and aggressive driving behavior. Further analytic research could provide deeper insight into how insomnia, cognitive function, mental health, and aggressive driving behavior evolve over time among taxi drivers. Second, the possibility of selection bias arose by relying on a non-probability sample of volunteers, and therefore may not fully represent the broader population of taxi drivers. This issue could be mitigated using a probability sampling method to ensure a more representative sample. Lastly, the average age of the subjects in this study was 59 years old, with 21.3% of the taxi drivers over 65 years old. However, since taxi drivers older than 65 years were not analyzed separately, caution is needed in interpreting the study results. Future long-term follow-up research is needed to confirm the association between aggressive driving behavior and its effects and taxi drivers aged 65 years or older.

## Figures and Tables

**Figure f2-epih-46-e2024085:**
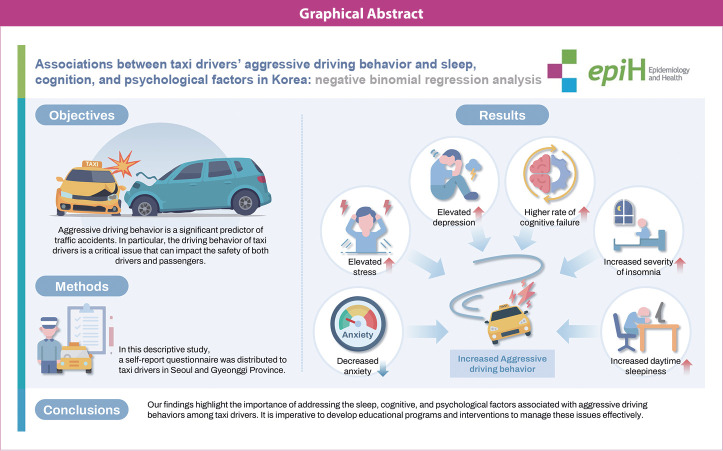


**Table 1.** General and driving-related characteristics of taxi drivers in Korea (n=992)

Characteristics	Categories	n (%) or mean±SD
Age (yr)		59.27±6.84
	40s	50 (5.0)
	50s	518 (52.2)
	60s	351 (35.4)
	70s	64 (6.5)
	≥80s	9 (0.9)
Sex	Male	975 (98.3)
	Female	17 (1.7)
BMI (kg/m^2^)		24.67±2.77
Education	<High school	195 (19.7)
	High school	661 (66.6)
	≥College or university	136 (13.7)
Marital status	Single	45 (4.6)
	Married with spouse	751 (75.7)
	Divorce	155 (15.6)
	Separation	41 (4.1)
No. of cohabiting family		1.60±1.08
Personal income/mo (10^4^ Korean won)	<100	30 (3.0)
	100-149	192 (19.4)
	150-199	299 (30.1)
	200-249	249 (25.1)
	250-299	159 (16.0)
	≥300	63 (6.4)
Smoking	Yes	461 (46.5)
	No	531 (53.5)
Drinking	Yes	724 (73.0)
	No	268 (27.0)
Hobbies	Yes	794 (80.0)
	No	198 (20.0)
Comorbidities	Yes	564 (56.8)
	No	428 (43.2)
Types of comorbidities (duplicate responses included)	HTN	353 (62.6)
	DM	208 (36.9)
	Digestive system disease	65 (11.5)
	Herniated intravertebral disc	44 (7.8)
	Arthritis	19 (3.4)
	Angina and myocardial infarction	26 (4.6)
	Cancer	17 (3.0)
	Stroke	15 (2.6)
	Liver disease	13 (2.3)
	Cataract	12 (2.1)
	Heart failure and arrhythmia	11 (1.9)
	Thyroid disease	7 (1.2)
	Hyperlipidemia	6 (1.1)
Type of taxi	Corporate	700 (70.6)
	Private	292 (29.4)
Driving experience (mo)		197.86±120.56
Driving hours (hr/day)		10.20±2.36
Driving distance (km/day)		223.28±67.18
No. of night shifts per fortnight		5.12±4.87
Car driving aids	Yes	472 (47.6)
	No	520 (52.4)
Types of car driving aids (duplicate responses included)	Lane departure warning/lane keep assist system	411 (87.1)
	Automatic emergency braking device	181 (38.3)
	Car forward collision warning system	217 (46.0)
	Car rear and sides collision warning system	349 (73.9)
	Highway driving assistance system	130 (27.5)
	Other	3 (0.6)

SD, standard deviation; BMI, body mass index; DM, diabetes mellitus; HTN, hypertension.

**Table 2.** The aggressive driving behaviors of taxi drivers in Korea (n=992)

Driving behavior questionnaire	Mean±SD
Total	13.76±11.47
Lapse (L)	3.46±3.48
Error (E)	3.31±3.16
Violation (V)	6.99±5.76
1. Attempt to overtake someone turning left (E)	0.95±0.99
2. Get into the wrong lane approaching a roundabout or a junction (L)	0.77±0.75
3. Miss “Give Way” signs (L)	0.48±0.71
4. Misread the signs, exit from a roundabout on the wrong road (L)	0.43±0.67
5. Fail to see pedestrians crossing (E)	0.37±0.57
6. Close following (V)	0.39±0.74
7. Forget where you left your car (L)	0.21±0.49
8. Queuing, nearly hit car in front (E)	0.39±0.58
9. Hit something when reversing (E)	0.36±0.53
10. Push in at last minute (V)	0.91±0.71
11. Turning right nearly hit cyclist (L)	0.40±0.62
12. Speed through lights (V)	0.69±0.86
13. Underestimate the speed of an oncoming vehicle (E)	0.41±0.65
14. Fail to check your rear-view mirror (L)	0.43±0.64
15. Aversion, indicate hostility (V)	0.46±0.64
16. Overtake a slow driver on the inside (V)	0.90±0.83
17. Attempt to drive away in third gear (E)	0.21±0.44
18. Switch on one thing, meaning the other (E)	0.29±0.51
19. Brake too quickly on a slippery road (E)	0.33±0.54
20. Intending to drive to destination A, instead drive to B (L)	0.37±0.57
21. Pull out, force your way out (V)	0.46±0.68
22. Race from lights (V)	0.30±0.60
23. Have no clear recollection of the road (L)	0.36±0.57
24. Get angry, give chase (V)	0.24±0.47
25. Sound horn to indicate your annoyance (V)	0.75±0.75
26. Disregard the speed limit on a motorway (V)	0.90±0.79
27. Disregard the speed limit on a residential road (V)	0.46±0.68
28. Stay in motorway lane knowing it will be closed (V)	0.52±0.77

SD, standard deviation.

**Table 3.** Sleep disorders, cognitive decline, and psychological factors in Korean taxi drivers (n=992)

Variable	Categories	n (%) or mean±SD
Sleep disorder	Insomnia severity	4.71±4.11
	No clinically significant insomnia	749 (75.5)
	Subthreshold insomnia	217 (21.9)
	Moderate insomnia	26 (2.6)
	Severe insomnia	0 (0.0)
	Daytime sleepiness	4.74±3.70
Cognitive decline	Total	1.54±2.20
	Subjective memory decline	
	Overall memory loss	0.70±0.86
	Daily life memory loss	0.84±1.61
	Cognitive failure	11.15±10.88
Psychological factors	Total	6.33±8.36
	Depression	2.05±2.91
	Anxiety	1.84±2.64
	Stress	2.44±3.15

SD, standard deviation.

**Table 4.** Sleep disorders, cognitive decline, and psychological factors affecting the aggressive driving behaviors of taxi drivers in Korea (n=992)

Variables	Estimate	SE	z	p> |z|	Rate ratio (95% CI)
Insomnia severity	0.027	0.003	8.347	<0.001	1.03 (1.02, 1.03)
Daytime sleepiness	0.051	0.004	12.949	<0.001	1.05 (1.04, 1.06)
Subjective memory decline	-0.008	0.005	-1.561	0.118	0.99 (0.98, 1.00)
Cognitive failure	0.033	0.002	19.805	<0.001	1.03 (1.03, 1.04)
Depression	0.018	0.008	2.269	0.023	1.02 (1.00, 1.03)
Anxiety	-0.046	0.009	-5.293	<0.001	0.95 (0.94, 0.97)
Stress	0.025	0.008	3.312	0.001	1.02 (1.01, 1.04)
Log_2_ likelihood: -34,738.48; AIC: 34,756; Theta: 2.446; SE: 0.066					

SE, standard error; CI, confidence interval; AIC, Akaike information criterion.
